# Age at type 2 diabetes onset and risk of dementia: The modifying role of genetic susceptibility and mitochondrial function

**DOI:** 10.1016/j.jnha.2026.100780

**Published:** 2026-01-17

**Authors:** Wanqing Dong, Qibin Yuan, Benrui Wu, Shiteng Gao, Yingyu Zhang, Ying Pan, Kaixin Zhou, Hongwei Jiang

**Affiliations:** a The First Affiliated Hospital, and College of Clinical Medicine of Henan University of Science and Technology, Luoyang, 471003, China; b Key Laboratory of Hereditary Rare Diseases of Health Commission of Henan Province, Luoyang, 471003, China; c Henan Key Laboratory of Rare Diseases, Luoyang, 471003, China; d Endocrinology and Metabolism Center, Luoyang, 471003, China; e The First People's Hospital of Shangqiu, Shangqiu, 476000, China; f National Laboratory of Biomacromolecules, Institute of Biophysics Chinese Academy of Sciences, Beijing, 100101, China; g College of Life Sciences, University of the Chinese Academy of Sciences, Beijing,101408, China; h Guangzhou National Laboratory, No. 9 XingDaoHuanBei Road, Guangzhou International Bio Island, Guangzhou, Guangdong Province, 510005, China; i Department of General Practice, Affiliated Kunshan Hospital of Jiangsu University, Suzhou, Jiangsu Province, 215300, China; j Kunshan Biomedical Big Data Innovation Application Laboratory, Jiangsu, 215300, China

**Keywords:** Age at diabetes onset, Dementia, Polygenic risk score, Mitochondrial DNA copy number

## Abstract

**Objectives:**

To assess dementia risk after incident type 2 diabetes (T2D) by age at diagnosis and evaluate modification by treatment, genetic susceptibility, and mitochondrial function.

**Design:**

Prospective 1:1 age- and sex-matched cohort study using inverse-probability-weighted Cox models.

**Setting:**

Kunshan Aging Research with E-health (KARE) cohort in China (2018–2024).

**Participants:**

42,514 adults without diabetes or dementia at baseline, including 21,257 incident T2D cases and 21,257 non-diabetic controls.

**Measurements:**

Outcomes were all-cause dementia, Alzheimer’s disease (AD), and vascular dementia (VaD) from linked medical records and annual examinations. T2D onset age was grouped as 45–54 years, 55–64 years, and persons 65 years and older. In genotyped participants (*n* = 14,455), a T2D polygenic risk score (PRS) and blood mitochondrial DNA copy number (mtDNA-CN) were examined.

**Results:**

Over a median 3.67 years, incident T2D was associated with higher risks of all-cause dementia (adjusted hazard ratio [AHR] 1.95, 95% CI 1.71–2.21), AD (2.21, 1.88–2.59), and VaD (1.57, 1.20–2.07). Glucose-lowering treatment was associated with lower dementia risk versus no treatment. Among patients aged 55–64 years, the low-PRS/low-mtDNA-CN subgroup had the highest AD risk (AHR 2.41, 95% CI 1.12–5.19).

**Conclusion:**

Age at T2D onset was associated with variation in dementia risk. Earlier diagnosis and treatment were associated with lower observed cognitive risk, while genetic susceptibility and mitochondrial function may inform individualised risk stratification.

## Highlights

• Incident T2D was associated with higher risks of all-cause dementia, AD, and VaD.
• Dementia risk heterogeneity was observed across T2D age-at-diagnosis groups.
• Glucose-lowering treatment initiation was associated with lower dementia risk.
• PRS and mtDNA-CN modified associations between incident T2D and dementia.
• Low PRS plus low mtDNA-CN showed the strongest AD-risk signal at ages 55–64 years.

## 1 Introduction

The global prevalence of type 2 diabetes (T2D) continues to rise, driven by population ageing and behavioural, environmental, and socioeconomic factors [1]. As of 2025, an estimated 589 million adults aged 18–79 are living with diabetes, over 90% of whom have T2D, with projections indicating this figure will reach 853 million by 2050 [2].This epidemic heightens the burden of age-related conditions such as cognitive impairment, posing major challenges for healthcare systems [3]. Substantial evidence indicates that T2D significantly increases the risk of dementia [4], with meta-analyses reporting a 1.25–1.91-fold elevated dementia risk in individuals with T2D compared to those without, including associations with both Alzheimer’s disease (AD) and vascular dementia (VaD) [5]. Importantly, this elevated risk is observed regardless of whether T2D is diagnosed in midlife or later [6]. As a modifiable metabolic condition, effective T2D management may thus reduce the burden of neurodegenerative diseases.

Despite the established link between T2D and dementia, it remains unclear whether age at diagnosis modifies this association. Emerging studies suggest that earlier onset of T2D may confer a higher risk of adverse outcomes [7]. For instance, Wang et al. found that individuals diagnosed before age 55 faced greater risks of dementia and stroke than those diagnosed at ≥65 [8], while Seo et al. reported a stepwise decline in dementia risk with each 10-year delay in T2D onset [9]. However, other findings challenge this notion. A 13-year follow-up study observed the highest dementia risk among those diagnosed at ≥65 [10]. Such inconsistencies leave unresolved whether age at T2D diagnosis is a critical modifier of dementia risk, thereby limiting targeted prevention efforts. Specifically, it remains uncertain whether prioritising glycaemic control in middle-aged adults would yield greater cognitive benefits than focusing on older populations. Moreover, most prior studies have treated T2D as a static exposure, overlooking the dynamic influence of diagnosis timing. Given that age at onset determines the cumulative duration of hyperglycaemia, it may better capture T2D’s long-term impact on brain health. However, prospective evidence addressing this question is scarce. Retrospective studies are prone to recall and selection biases, limiting their ability to characterise age-gradient effects. Clarifying the relationship between age at T2D onset and cognitive and survival outcomes using prospective data is essential to understand underlying mechanisms, identify high-risk subgroups, and inform precision prevention strategies.

Notably, although T2D is generally linked with higher dementia risk, substantial interindividual heterogeneity exists—many patients never develop dementia [11]. This suggests the involvement of unmeasured modifiers. Two key biological characteristics—genetic susceptibility and mitochondrial function—may shape individual vulnerability to T2D-related neurodegeneration. The polygenic risk score (PRS) for T2D quantifies genetic predisposition and has been associated with earlier T2D onset and increased risk of AD and related dementias (ADRD) in UK Biobank studies [12]. Dybjer et al. reported that T2D PRS was especially predictive of VaD [13], consistent with the stronger association between T2D and vascular rather than other dementia subtypes [14]. In the US Million Veteran Program, associations between a T2D PRS and dementia outcomes were assessed across race/ethnicity strata (EUR, AFR, and HIS), with statistically significant associations observed mainly in the EUR stratum [15]. Conversely, other studies have found no significant association between T2D PRS and dementia [16].

Additionally, mitochondrial dysfunction may mediate T2D’s neurological impact via impaired energy metabolism and oxidative stress. In animal models, mitochondrial impairment promotes reactive oxygen species (ROS) accumulation and neuroinflammation, fostering amyloidogenesis and neurodegeneration [17]. In humans, mitochondrial oxidative phosphorylation supplies the majority of cellular energy, making high-demand organs like the brain particularly susceptible to dysfunction [17]. While direct assessment of mitochondrial function in large studies is challenging, blood mitochondrial DNA copy number (mtDNA-CN) serves as a validated proxy. Studies have shown that mtDNA-CN declines with age [18] and is inversely associated with dementia and mortality risk [[19], [20], [21]]. Thus, both T2D PRS and mtDNA-CN may independently or synergistically modulate the relationship between T2D and neurodegeneration. Yet, no study to date has systematically examined their modifying or interactive effects on T2D-associated cognitive decline, particularly during the early post-diagnosis period—a potentially critical window for intervention.

To address these gaps, we used data from a large prospective community cohort in Eastern China to examine the association between newly diagnosed T2D and risks of all-cause dementia, AD, VaD. We evaluated whether age at diagnosis modifies these associations and explored the roles of T2D PRS and mtDNA-CN as potential effect modifiers. This study aims to provide empirical evidence to clarify mechanisms, identify high-risk subgroups, and inform tailored prevention strategies.

## 2 Methods

### 2.1 Study design and population

This study was based on the Kunshan Aging Research with E-health (KARE) cohort, a bidirectional prospective study initiated in Kunshan, Jiangsu Province, China. Details of the cohort design and follow-up procedures have been described elsewhere [22]. As of 2024, the cohort included 138,199 participants. All participants provided informed consent for use of health data and residual blood samples during annual health examinations. We identified incident T2D cases diagnosed after 1 January 2018, excluding individuals with: missing diagnosis dates (*n* = 1); a single T2D diagnosis record (*n* = 13,284); T2D diagnosed before 2018 (*n* = 25,859); pre-2018 dementia (*n* = 823) or death (*n* = 390); non-T2D diabetes (ICD-10 E10, O24.4, O24.9, E13, E14) (*n* = 886); missing covariates (*n* = 1227); or age <45 years at diagnosis (*n* = 702). This yielded 21,257 incident T2D cases. Controls were matched 1:1 by age and sex from contemporaneous participants without diabetes or dementia, resulting in 42,514 individuals.Follow-up began at the date of first T2D evidence for cases and at the health check-up date in the corresponding year for controls. Participants were followed via annual health examinations and linkage to the municipal electronic medical record (EMR) system until dementia onset, death, loss to follow-up, or 24 August 2024.

### 2.2 Definition of incident T2D

T2D was defined as meeting either of the following: (1) ICD-10 diagnosis codes E11; (2) two or more fasting plasma glucose (FPG) measurements ≥7.0 mmol/L. Diagnosis date was assigned as the first occurrence. Only new-onset cases after 1 January 2018 were included to minimise recall bias.

### 2.3 Outcome ascertainment

The primary outcomes were all-cause dementia, AD, VaD. AD was defined by ICD-10 codes F00 or G30, or use of anti-dementia drugs (e.g., cholinesterase inhibitors or memantine). VaD was defined by F01 or I67.3; other dementia types by F02, F03, or A81.0. Dementia diagnoses were obtained from EMRs and validated by neurologists according to established procedures [23,24].

### 2.4 Covariate assessment

Baseline data were extracted from health examinations, including age, sex, body mass index (BMI), systolic and diastolic blood pressure, FPG, and lipid profiles (total cholesterol, triglycerides, LDL-C, HDL-C). Comorbidities were defined by ICD-10 codes: cerebrovascular disease (I60–I69), ischaemic heart disease (I20–I25), heart failure (I50), sleep disorders (G47, F51), and depression (F31–F34). Medication use was obtained from prescription records.

### 2.5 Genotyping and PRS construction

Genotyping was performed using the Affymetrix CAS array tailored for East Asian populations, with protocols for genotyping, quality control, and imputation previously described [25]. Variants and samples were excluded if SNP call rate < 0.98, minor allele frequency < 0.05, Hardy–Weinberg equilibrium *P* < 1 × 10^−^⁶; or if sample call rate < 0.90, sex mismatch, abnormal heterozygosity, or close relatedness (PI-HAT > 0.9). Phasing and imputation were conducted using SHAPEIT 4.2.2 [26] and Beagle 5.4 [27] with the 1000 Genomes Project high-coverage reference panel [28], excluding variants with INFO < 0.3.The T2D PRS was based on genome-wide significant SNPs (*P* < 5 × 10^−^⁸) from the BioBank Japan GWAS [29]. Using the *bigsnpr* R package [30] and the clumping and thresholding (C + T) method [31] (r² < 0.2, 500 kb window), 245 independent SNPs were selected. The PRS was computed as a weighted sum of risk alleles (using GWAS β coefficients) and standardised.

### 2.6 Estimation of mtDNA-CN

mtDNA-CN was estimated from raw CAS array intensity data following the MitoPipeline [32] and AutoMitoC [33,34] workflows. After GC correction using PennCNV [35,36], outlier samples were excluded based on log R ratio (LRR) variability. Principal component analysis (PCA) of autosomal SNPs identified 272 PCs accounting for technical variation, which were used to adjust mitochondrial LRR. The first PC from adjusted mitochondrial SNPs was used as the mtDNA-CN estimate. Direction was confirmed based on its inverse correlation with age.

### 2.7 Statistical analysis

Incident T2D cases were categorised by age at diagnosis: 45–54, 55–64, and ≥65 years. Continuous and categorical variables were compared using ANOVA/Kruskal–Wallis and chi-squared tests, respectively. Inverse probability weighting (IPW) Cox models estimated adjusted hazard ratios (HRs) and 95% confidence intervals (CIs) for associations between T2D and outcomes. Propensity scores were derived from logistic regression including age (per 10 years), sex, BMI, FPG, lipid profiles, blood pressure, and comorbidities. Average treatment effect (ATE) weights were applied and truncated at the 99th percentile for stability. Covariate balance was assessed using standardised mean differences (SMDs <0.1) and Kolmogorov–Smirnov tests.Subgroup analyses stratified T2D patients by treatment status (treated vs untreated), PRS (above vs below median), and mtDNA-CN (above vs below median). Joint stratification combined PRS and mtDNA-CN into four exposure groups. Analyses were performed in R v4.3.1 using two-sided tests with α = 0.05.

## 3 Results

### 3.1 Baseline characteristics

A total of 42,514 participants were included, comprising 21,257 individuals with newly diagnosed T2D and 21,257 matched controls. The median follow-up duration was 3.67 years. Compared with controls, T2D patients had higher baseline BMI, fasting glucose, triglycerides, LDL-C, and systolic blood pressure. They also had higher prevalence of ischaemic heart disease, cerebrovascular disease, heart failure, depression, and sleep disorders (Table 1). Among T2D patients, increasing age at diagnosis was associated with lower fasting glucose levels and a higher likelihood of glucose-lowering medication use (Table 2).

**Table 1:** Baseline demographic and clinical characteristics of participants with newly diagnosed type 2 diabetes and non-diabetes controls.

Variable	Total (*n* = 42,514)	Non-diabetes (*n* = 21,257)	newly diagnosed T2D (*n* = 21,257)	*P*-value
Age, years [median, IQR]	65.19 [58.82,70.18]	65.19 [58.81,70.18]	65.19 [58.82, 70.18]	0.99
Male, *n* (%)	19,428 (45.70)	9714 (45.70)	9714 (45.70)	1.00
Female, *n* (%)	23,086 (54.30)	11,543(54.30)	11,543 (54.30)	
Follow-up (years), [median, IQR]	3.67 [1.85,5.17]	3.86 [1.94,5.14]	3.46 [1.69, 5.21]	<0.01
All-cause dementia, *n* (%)	1808 (4.25)	731 (3.44)	1077 (5.07)	<0.01
Alzheimer’s disease (AD), *n* (%)	1152(2.71)	438 (2.06)	714 (3.36)	<0.01
Vascular dementia (VD), *n* (%)	379(0.89)	165 (0.78)	214 (1.01)	0.01
All-cause mortality, *n* (%)	546 (1.28)	203 (0.95)	343 (1.61)	<0.01
BMI, (median [IQR])	24.51 [22.48,26.71]	24.03 [22.03,26.17]	24.98 [22.96, 27.23]	<0.01
LDL-C, mmol/L [median, IQR]	2.80 [2.30,3.39]	2.80 [2.30,3.30]	2.8 3 [2.30, 3.40]	<0.01
HDL-C, mmol/L [median, IQR]	1.30 [1.10,1.57]	1.35 [1.13,1.60]	1.30 [1.10, 1.50]	<0.01
TC, mmol/L [median, IQR]	4.85 [4.20,5.50]	4.82 [4.25,5.50]	4.86 [4.20, 5.54]	0.49
TG, mmol/L [median, IQR]	1.40 [1.00,2.00]	1.30 [0.96,1.86]	1.50 [1.08, 2.18]	<0.01
FG, mmol/L (median [IQR])	5.53 [5.10,6.30]	5.20 [4.90,5.60]	6.21 [5.49, 7.14]	<0.01
SBP, mmHg (median [IQR])	136 [125,148]	135[124,147]	137.0 [125, 149]	<0.01
DBP, mmHg (median [IQR])	81 [75,88]	81 [74,88]	81 [75, 88]	<0.01
Ischaemic heart disease, *n* (%)	8790 (20.68)	3201 (15.06)	5589 (26.29)	<0.01
Heart failure, *n* (%)	2776 (6.53)	1001 (4.71)	1775 (8.35)	<0.01
Sleep disorders, *n* (%)	5647 (13.28)	2172 (10.22)	3475 (16.35)	<0.01
Cerebrovascular disease, *n* (%)	12,518 (29.44)	4910 (23.10)	7608 (35.79)	<0.01
Depression, *n* (%)	1120 (2.63)	389(1.83)	731 (3.44)	<0.01
Antidiabetic medications, *n* (%)	9494 (22.33)	0(0.0)	9494 (44.66)	<0.01

Values are presented as median [interquartile range] or n (%). P-values compare the non-diabetes group and the newly diagnosed type 2 diabetes group.

Abbreviations: T2D, type 2 diabetes; BMI, body mass index; LDL-C, low-density lipoprotein cholesterol; HDL-C, high-density lipoprotein cholesterol; TC, total cholesterol; TG, triglycerides; FG, fasting glucose; SBP, systolic blood pressure; DBP, diastolic blood pressure.

**Table 2:** Baseline characteristics of participants with newly diagnosed type 2 diabetes across different age-at-diagnosis groups.

Variable	Age 45–54 years (*n* = 3263)	Age 55–64 years (*n* = 7116)	Age ≥65 years (*n* = 10878)
Age, years [median, IQR]	51.64 [49.39, 53.46]	61.09 [58.27, 63.34]	70.01 [67.24, 74.21]
Male, *n* (%)	1337 (40.97)	3211 (45.12)	5166 (47.49)
Female, *n* (%)	1926 (59.03)	3905 (54.88)	5712 (52.51)
Follow-up (years), [median, IQR]	4.26 [2.65, 5.62]	4.00 [2.06, 5.46]	2.88 [1.25, 4.88]
All-cause dementia, *n* (%)	72 (2.21)	264 (3.71)	741 (6.81)
Alzheimer’s disease (AD), *n* (%)	40 (1.23)	165 (2.32)	509 (4.68)
Vascular dementia (VD), *n* (%)	5 (0.15)	52 (0.73)	157 (1.44)
All-cause mortality, *n* (%)	3 (0.09)	34 (0.48)	306 (2.81)
BMI, (median [IQR])	25.08 [23.08, 27.38]	24.89 [22.93, 27.12]	25.02 [22.95, 27.24]
LDL-C, mmol/L [median, IQR]	2.90 [2.40, 3.42]	2.90 [2.34, 3.50]	2.80 [2.20, 3.39]
HDL-C, mmol/L [median, IQR]	1.23 [1.04, 1.47]	1.30 [1.10, 1.52]	1.30 [1.10, 1.50]
TC, mmol/L [median, IQR]	4.89 [4.29, 5.52]	4.90 [4.25, 5.60]	4.80 [4.18, 5.50]
TG, mmol/L [median, IQR]	1.60 [1.13, 2.49]	1.50 [1.09, 2.20]	1.46 [1.04, 2.10]
FG, mmol/L (median [IQR])	6.37 [5.50, 7.40]	6.30 [5.50, 7.27]	6.19 [5.40, 7.00]
SBP, mmHg (median [IQR])	131 [120, 142]	134 [124, 146]	140 [128, 152]
DBP, mmHg (median [IQR])	83 [77, 90]	82 [76, 88]	81.00 [74, 88]
Ischaemic heart disease, *n* (%)	244 (7.48)	1796 (25.24)	3549 (32.63)
Heart failure, *n* (%)	56 (1.72)	487 (6.84)	1232 (11.33)
Sleep disorders, *n* (%)	220 (6.74)	1209 (16.99)	2046 (18.81)
Cerebrovascular disease, *n* (%)	394 (12.07)	2524 (35.47)	4690 (43.11)
Depression, *n* (%)	36 (1.10)	236 (3.32)	459 (4.22)
Antidiabetic medications, *n* (%)	1896 (58.11)	3488 (49.02)	4110 (37.78)

Age groups refer to age at the time of type 2 diabetes diagnosis.

Values are presented as median [interquartile range] or *n* (%).

Abbreviations: T2D, type 2 diabetes; BMI, body mass index; LDL-C, low-density lipoprotein cholesterol; HDL-C, high-density lipoprotein cholesterol; TC, total cholesterol; TG, triglycerides; FG, fasting glucose; SBP, systolic blood pressure; DBP, diastolic blood pressure.

### 3.2 Association between T2D and risks of dementia and mortality

After adjustment for covariates, incident T2D was associated with significantly elevated risks of all-cause dementia (adjusted HR [AHR] = 1.95; 95% CI: 1.71–2.21), AD (AHR = 2.21; 1.88–2.59), VaD (AHR = 1.57; 1.20–2.07), compared to non-diabetic controls. In the 45–54 age group, incident T2D significantly increased the risk of AD (AHR = 2.08; 95% CI: 1.02–4.23), while risks of all-cause dementia and VaD were not significantly different. In the 55–64 group, T2D was associated with higher risks of all-cause dementia (AHR = 1.67), AD (AHR = 1.92), but not VaD. In the ≥65 group, incident T2D significantly increased the risk of all-cause dementia (AHR = 2.07), AD (AHR = 2.34), VaD (AHR = 2.34) (Table 3).

**Table 3:** Association of newly diagnosed type 2 diabetes and treatment status with risks of dementia, by age at diagnosis.

Group	Newly diagnosed T2D (vs. non-diabetes)				Treated T2D (vs. untreated T2D)
	Overall T2D (*n* = 21,257)		Untreated T2D (*n* = 11,763)	Treated T2D (*n* = 9494)	
	Events, *n* (%)	AHR (95% CI)	AHR(95% CI)	AHR(95% CI)	AHR(95% CI)
All-cause dementia					
Total	1077(5.07%)	1.95(1.71−2.21)	1.97(1.74−2.24)	1.25(1.06−1.48)	0.59(0.50−0.67)
45−54 yrs	72(2.21%)	1.70(1.00−2.88)	1.72(1.02−2.90)	1.33(0.72−2.45)	0.37(0.19−0.72)
55−64 yrs	264(3.71%)	1.67(1.25−2.24)	1.71(1.28−2.28)	1.78(1.29−2.45)	0.85(0.66−1.10)
≥65 yrs	741(6.81%)	2.07(1.78−2.40)	2.07(1.78−2.41)	1.04(0.84−1.27)	0.51(0.44−0.61)
Alzheimer’s disease					
Total	714(3.36%)	2.21(1.88−2.59)	2.22(1.89−2.61)	1.42(1.16−1.74)	0.62(0.53−0.73)
45−54 yrs	40(1.23%)	2.08(1.02−4.23)	2.05(1.02−4.13)	0.88(0.41−1.91)	0.19(0.07−0.50)
55−64 yrs	165(2.32%)	1.92(1.33−2.77)	1.90(1.32−2.74)	1.81(1.21−2.72)	0.88(0.64−1.23)
≥65 yrs	509(4.68%)	2.34(1.81−3.04)	2.35(1.95−2.82)	1.31(1.03−1.67)	0.57(0.47−0.69)
Vascular dementia					
Total	214(1.01%)	1.57(1.20−2.07)	1.63(1.24−2.15)	0.80(0.56−1.14)	0.45(0.33−0.61)
45−54 yrs	5(0.15%)	0.99(0.26−3.77)	0.90(0.22−3.66)	0.37(0.07−1.89)	0.21(0.02−1.98)
55−64 yrs	52(0.3%)	1.29(0.70−2.37)	1.36(0.75−2.44)	1.35(0.71−2.58)	0.69(0.38−1.26)
≥65 yrs	157(1.44%)	2.34(1.95−2.80)	1.79(1.30−2.48)	0.69(0.44−1.06)	0.39(0.27−0.56)

Adjusted for age (continuous), sex, body mass index (BMI), low-density lipoprotein cholesterol (LDL-C), high-density lipoprotein cholesterol (HDL-C), total cholesterol (TC), triglycerides (TG), fasting glucose (FG), systolic blood pressure (SBP), diastolic blood pressure (DBP), ischaemic heart disease, heart failure, sleep disorders, cerebrovascular disease, and depression.

Abbreviations: AHR = adjusted hazard ratio; CI = confidence interval. T2D = type 2 diabetes. Numbers of participants in each stratum were as follows: total (*n* = 21,257), age 45–54 years (*n* = 3263), age 55–64 years (*n* = 7117), and age ≥65 years (*n* = 10,877).

### 3.3 Diabetes treatment and risk modification

Among participants with newly diagnosed T2D, glucose-lowering treatment was associated with a lower risk of dementia (Table 3). Compared with untreated patients, treated individuals had a 41% lower risk of all-cause dementia (AHR = 0.59), with similar reductions observed for AD (AHR = 0.62) and VaD (AHR = 0.45). The protective association was generally consistent across age groups and most pronounced among those diagnosed at 45–54 years (Table 3).

### 3.4 Stratified analyses by PRS and mtDNA-CN

Among 14,455 participants with genotype data, T2D PRS was positively associated with incident T2D (OR = 1.28; 95% CI: 1.24–1.32; *P* < 0.01), while mtDNA-CN was inversely associated (OR = 0.97; 95% CI: 0.96–0.98; *P* < 0.01). Stratified analyses by T2D PRS and mtDNA-CN indicated that the associations of newly diagnosed T2D with all-cause dementia and AD were more pronounced among individuals with low PRS or low mtDNA-CN, whereas the corresponding associations were weaker among those with high PRS or high mtDNA-CN. In contrast, no consistent pattern was observed for VaD across PRS and mtDNA-CN strata (Table 4). Among participants with newly diagnosed T2D, joint stratification using the high PRS and high mtDNA-CN subgroup as the reference showed that only individuals aged 55–64 years with both low PRS and low mtDNA-CN had a significantly increased risk of AD (AHR = 2.41, 95% CI: 1.12–5.19), whereas no significant risk elevation was observed for other PRS–mtDNA-CN combinations across age groups (Table 5).

**Table 4:** Hazard ratios for dementia among participants with newly diagnosed type 2 diabetes, stratified by T2D polygenic risk score, mitochondrial DNA copy number, and age at diagnosis.

Group	Low T2D PRS		High T2D PRS		T2D **mtDNA-CN** Low		T2D **mtDNA-CN** High	
	Case	AHR (95% CI)	Case	AHR (95% CI)	Case	AHR (95% CI)	Case	AHR (95% CI)
All-cause dementia								
Total	225	1.71(1.38−2.12)	202	1.38(1.10−1.73)	224	1.54(1.23−1.92)	203	1.57(1.26−1.96)
45–54 yrs	8	1.62(0.78−3.37)	7	0.73(0.25−2.12)	9	1.42(0.62−3.27)	6	0.96(0.40−2.29)
55–64 yrs	53	2.04(1.25−3.31)	41	1.13(0.71−1.80)	49	1.75(1.06−2.89)	45	1.53(0.92−2.55)
≥65 yrs	14	1.62(1.26−2.08)	154	1.55(1.19−2.02)	166	1.50(1.16−1.94)	152	1.67(1.30−2.16)
Alzheimer’s disease								
Total	140	2.02(1.53−2.66)	130	1.58(1.18−2.12)	151	1.95(1.48−2.58)	119	1.68(1.25−2.25)
45–54 yrs	3	1.71(0.65–4.51)	5	1.01(0.23−4.38)	0	1.87(0.61−5.72)	3	0.95(0.28−3.17)
55–64 yrs	41	3.38(1.88−6.10)	22	1.20(0.63−2.29)	33	2.62(1.38−4.98)	30	2.15(1.12−4.14)
≥65 yrs	96	1.70(1.23−2.34)	103	1.75(1.26−2.43)	113	1.78(1.31−2.43)	86	1.67(1.19−2.33)
Vascular dementia								
Total	57	1.21(0.81−1.82)	46	1.01(0.64−1.59)	51	0.97(0.63−1.49)	52	1.26(0.83−1.92)
45–54 yrs	1	0.16(0.02−1.29)	0	–	5	0.16(0.02−1.29)	0	–
55–64 yrs	10	0.74(0.30−1.81)	10	0.88(0.37−2.06)	11	0.83(0.37−1.89)	9	0.77(0.29−1.99)
≥65 yrs	46	1.47(0.91−2.38)	36	1.21(0.70−2.08)	39	1.13(0.68−1.90)	43	1.55(0.95−2.53)

Adjusted for age (continuous), sex, body mass index (BMI), low-density lipoprotein cholesterol (LDL-C), high-density lipoprotein cholesterol (HDL-C), total cholesterol (TC), triglycerides (TG), fasting glucose (FG), systolic blood pressure (SBP), diastolic blood pressure (DBP), ischaemic heart disease, heart failure, sleep disorders, cerebrovascular disease, and depression.

Each subgroup was compared with non-diabetes controls. “–” indicates that the estimate was not reported because of insufficient events within the corresponding subgroup, resulting in unstable model estimates.AHR = adjusted hazard ratio; CI = confidence interval; T2D, type 2 diabetes; PRS = polygenic risk score.

**Table 5:** Dementia risk among participants with newly diagnosed type 2 diabetes, stratified by T2D polygenic risk score, mitochondrial DNA copy number, and age at diagnosis, with the high PRS and high mtDNA-CN group as the reference.

Group	T2D							
	H-PRS & H-mtDNA-CN		L-PRS & H-mtDNA-CN		H-PRS & L-mtDNA-CN		L-PRS& L-mtDN CN	
	Case	AHR	Case	AHR (95% CI)	Case	AHR (95% CI)	Case	AHR (95% CI)
All-cause dementia								
Total	91	reference	112	1.22(0.93−1.62)	111	1.12(0.85−1.48)	113	1.13(0.86−1.50)
45–54 yrs	2	reference	4	1.94(0.34−11.20)	5	2.71(0.52−14.17)	4	2.96(0.54−16.35)
55–64 yrs	19	reference	26	1.35(0.74−2.44)	22	1.20(0.65−2.23)	27	1.53(0.84−2.77)
≥65 yrs	70	reference	82	1.12(0.81−1.54)	84	1.02(0.74−1.41)	82	0.97(0.70−1.34)
Alzheimer’s disease								
Total	59	reference	60	1.01(0.70−1.44)	71	1.11(0.78−1.56)	80	1.27(0.91−1.79)
45–54 yrs	1	reference	2	1.96(0.16−23.69)	4	4.21(0.47−38.09)	1	1.63(0.10−26.61)
55–64 yrs	10	reference	20	2.04(0.95−4.39)	12	1.29(0.55−3.00)	21	2.41(1.12−5.19) *
≥65 yrs	48	reference	38	0.74(0.48−1.33)	55	0.95(0.65−1.41)	58	1.03(0.70−1.51)
Vascular dementia								
Total	20	reference	32	1.60(0.91−2.82)	26	1.16(0.65−2.08)	25	1.05(0.58−1.90)
45–54 yrs	0	reference	0	–	0	–	1	–
55–64 yrs	4	reference	5	0.86(0.22−3.36)	6	1.11(0.31−4.06)	5	0.86(0.22−3.36)
≥65 yrs	16	reference	27	1.70(0.91−3.18)	20	1.08(0.56−2.10)	19	0.96(0.49−1.89)

Adjusted for age (continuous), sex, body mass index (BMI), low-density lipoprotein cholesterol (LDL-C), high-density lipoprotein cholesterol (HDL-C), total cholesterol (TC), triglycerides (TG), fasting glucose (FG), systolic blood pressure (SBP), diastolic blood pressure (DBP), ischaemic heart disease, heart failure, sleep disorders, cerebrovascular disease, and depression.

Participants with newly diagnosed type 2 diabetes were jointly stratified by type 2 diabetes polygenic risk score (T2D PRS: high vs low) and mitochondrial DNA copy number (mtDNA-CN: high vs low), forming four subgroups. The subgroup with high PRS and high mtDNA-CN was used as the reference group for all comparisons. “–” Indicates analysis not performed due to zero outcome events.AHR = adjusted hazard ratio; CI = confidence interval; T2D = type 2 diabetes; PRS = Polygenic risk score; mtDNA-CN = Mitochondrial DNA copy number; L-PRS& L-mtDN CN = Low PRS& Low mtDN CN; L-PRS & H-mtDNA-CN = Low PRS & High mtDNA-CN; H-PRS & L-mtDNA-CN =High PRS & Low mtDNA-CN; H-PRS & H-mtDNA-CN=High PRS & High mtDNA-CN. *Statistically significant at P < 0.05.

## 4 Discussion

Using prospective follow-up data from the KARE cohort, we investigated the associations between incident T2D and subsequent risks of dementia and its subtypes, and further evaluated effect modification by age at diagnosis, glucose-lowering treatment, genetic susceptibility (T2D PRS), and mitochondrial function (mtDNA-CN). Overall, incident T2D was associated with elevated risks of all-cause dementia, AD, and VaD, with marked heterogeneity across age-at-diagnosis groups. In addition, individuals who initiated glucose-lowering treatment promptly after diagnosis had lower dementia risks. Notably, among individuals aged 55–64 years, combined stratification by genetic background and mitochondrial status indicated substantial inter-individual variation: T2D patients with both low PRS and low mtDNA-CN exhibited a stronger signal for increased AD risk. Collectively, these findings suggest that T2D-related cognitive risk assessment should jointly consider the “metabolic-burden trajectory” captured by age at diagnosis, early treatment, and multidimensional biomarkers reflecting biological vulnerability.

The magnitude of the observed excess dementia risk associated with incident T2D was broadly consistent with prior meta-analytic evidence on T2D-related dementia risk elevations [5,10]. This concordance supports the view of T2D as an important, modifiable risk factor for dementia and further implies that effective management may be important for long-term brain health during critical periods of metabolic disturbance accumulation, particularly during midlife [8]. Importantly, because our analysis focused on a relatively short follow-up window after “incident T2D”, the findings are best interpreted as epidemiological evidence that, once individuals first meet clinical diagnostic thresholds for T2D, a detectable increase in dementia risk is already apparent within the ensuing years. This provides support for early risk identification and a potential window for timely intervention.

We observed clear differences in T2D-associated dementia risk by age at diagnosis. Among persons 65 years and older, more definitive risk elevations were observed for all-cause dementia, AD, and VaD; in the 45–54-year group, an increased signal for AD was also apparent; whereas in the 55–64-year group, the relative risk magnitude appeared lower. It is important to emphasise that these “non-monotonic” differences reflect variation in relative risk (HRs) across age strata rather than changes in absolute dementia risk, which rises steadily with advancing age in the general population [37]. Several non-mutually exclusive mechanisms may contribute to such heterogeneity. First, younger-onset T2D may indicate a more severe metabolic phenotype (more pronounced dysglycaemia at diagnosis and faster progression) [38], thereby yielding clearer cognitive-risk contrasts within the same observation window. Second, with increasing age, rising baseline dementia risk among same-age comparators may statistically “dilute” relative differences. Third, age at diagnosis marks the time of clinical recognition rather than the true onset of dysmetabolism [39], and different diagnosis ages may correspond to distinct disease stages or metabolic trajectories. Fourth, individuals diagnosed in midlife may be more likely to experience cardiovascular events or death before dementia manifests, introducing competing risks and survivor bias and leading to complex age-stratified patterns. Together, these considerations may shape the heterogeneous landscape linking age at T2D diagnosis to dementia-related relative risks.

Given the limited mean follow-up, our findings should not be simplistically attributed to dementia being “directly caused” within a short period by the accumulation of classic microvascular or macrovascular complications. A more plausible interpretation is that T2D and dementia share upstream metabolic pathology that develops over long periods, including IR, chronic low-grade inflammation, endothelial dysfunction, and disturbed energy metabolism [[40], [41], [42]]. Clinically, T2D diagnosis typically occurs after years of metabolic abnormalities; such long-standing dysregulation may already have exerted sustained effects on brain tissue, the cerebral vasculature, and the neuroimmune microenvironment prior to clinical recognition. Accordingly, “incident T2D” may be viewed as a sentinel event indicating that metabolic stress has reached a clinical threshold, and the subsequent rise in dementia risk may reflect an already heightened neurodegenerative vulnerability trajectory rather than the isolated short-term effect of hyperglycaemia. Consistent with this, prior work suggests that metabolic dysregulation may begin influencing the CNS early in the disease course—potentially before overt vascular complications are fully established [41]. Impaired insulin signalling, altered glucose metabolism, and accompanying inflammatory and oxidative stress responses are biologically linked to AD-related pathways [40] and may promote Aβ- and tau-related pathological changes at relatively early stages [43]. Thus, within the timeframe of our study, the association between incident T2D and increased dementia risk is best interpreted as an epidemiological signal aligned with shared metabolic–neurodegenerative mechanisms.

We further observed that, compared with untreated individuals, prompt glucose-lowering treatment was associated with substantially lower risks of dementia across subtypes, with broadly consistent patterns across age strata and more pronounced trends among those diagnosed at younger ages. This accords with existing evidence suggesting that improving glycaemic control and broader metabolic management may mitigate neurodegenerative risk [[8], [9], [10]]. Nevertheless, in real-world settings, “treatment” is likely to capture not only pharmacological effects but also healthcare access, health behaviours, follow-up adherence, and comorbidity management. A more cautious interpretation is therefore that timely treatment is associated with lower dementia risk, warranting further investigation in studies with stronger causal inference. In conjunction with external evidence, some trials and pharmacoepidemiological data have suggested that SGLT2i and GLP-1RA may be associated with lower dementia risk [44,45], providing direction for future work with stronger causal inference.

Our study also indicates that PRS and mtDNA-CN modify the T2D–dementia association. Under PRS stratification, T2D patients with low genetic susceptibility (low PRS) still exhibited more evident elevations in all-cause dementia and AD risk. Using peripheral blood leukocyte mtDNA-CN as a proxy for systemic mitochondrial quantity and functional status, mitochondrial function similarly modified the association between T2D and dementia. Of particular note, in the 55–64-year group, T2D patients characterised by both low PRS and low mtDNA-CN showed a stronger signal for increased AD risk, indicating greater heterogeneity in cognitive vulnerability within this age range. Beyond energy supply, mitochondria play essential roles in neuronal homeostasis and apoptotic regulation [46]. The “mitochondrial cascade hypothesis” posits that impaired mitochondrial metabolism may be an upstream driver of AD pathology [47,48]. Peripheral blood mtDNA-CN partially reflects systemic mitochondrial quantity and functional state [49,50]. Prior studies have reported that reduced mtDNA copy number is associated with increased AD risk [51], and higher mtDNA copy number in blood has been linked to lower dementia risk [19]. Multiple prospective studies have found that lower mtDNA-CN is associated with cognitive decline or higher dementia incidence [21,52], and is inversely associated with post-mortem brain Aβ and tau pathology burden [53]. Lower peripheral mtDNA-CN has been reported among individuals with AD or cognitive impairment compared with healthy controls [33,54], and UK Biobank analyses suggest higher mtDNA-CN is associated with reduced incidence of neurodegenerative diseases [55]. MR studies also support a potential causal role of low mtDNA-CN in AD-related biomarker abnormalities and dementia development [34,56]. It should be noted, however, that mtDNA-CN may be influenced by compensatory mitochondrial biogenesis and by variations in cellular composition and inflammatory status; accordingly, in some studies and specific subgroups, higher mtDNA indices may coexist with higher pathological burden or poorer cognitive phenotypes [56,57]. Against this evidence base, our results provide additional population-level clues that insufficient mitochondrial reserve may amplify T2D-related cognitive risk, especially in individuals with low genetic susceptibility who may represent a more “environment-driven” diabetes phenotype. This finding highlights mitochondrial function as a potentially relevant biological correlate of T2D-related cognitive risk, warranting further investigation in mechanistic and interventional studies. Future risk assessment and precision prevention may need to move beyond single metabolic indicators by integrating genetic background and mitochondrial-function metrics, which may reflect “metabolic resilience,” into comprehensive stratification frameworks.

The risk elevation observed for VaD was comparatively modest. Given that VaD is often linked to long-term accumulation of cerebrovascular injury [41], clinically diagnosable VaD phenotypes may become more apparent over longer time horizons; thus, within a relatively short follow-up window, VaD effects may not yet be fully “expressed”. In addition, the number of VaD events was relatively limited, which may reduce estimation precision. Moreover, individuals with T2D are at higher risk of stroke and other fatal cardiovascular outcomes, and such competing events may occur before potential VaD onset, thereby attenuating observed VaD incidence and influencing risk estimates. Future studies with longer follow-up, more events, and explicit competing-risk frameworks may help clarify long-term trends in the associations between T2D and dementia subtypes.

Taken together, our findings underscore the clinical importance of early cognitive-risk assessment and multidimensional, stratified management among patients with T2D. Although current interventions primarily target glycaemic control, our results suggest that age, genetic background, and mitochondrial function should also be considered for integrated assessment and tailored intervention. WHO and related guidance emphasise the reduction of modifiable risk factors as a key strategy to address dementia burden [58,59]. For example, for early-onset T2D, more intensive glycaemic management and cardiovascular risk control may be warranted. For individuals with low genetic risk but metabolically vulnerable profiles (low mtDNA-CN), closer monitoring of cognitive changes may be particularly important. Overall, incorporating multidimensional information into individualised risk stratification may facilitate early identification of high-risk groups and the development of lifelong, precision intervention strategies to minimise the burden of diabetes-related dementia.

This study has several strengths. Leveraging a large, well-characterised prospective cohort, with annual standardised health examinations and linkage to citywide EMRs, enhanced the accuracy of identifying incident T2D and capturing dementia outcomes. We applied strict 1:1 matching by age and sex and avoided exposure misclassification due to controls developing diabetes during follow-up. Conducted in a Chinese population and analysing dementia subtypes, the study complements evidence from non-Western populations and further explores inter-individual heterogeneity attributable to genetic factors and mitochondrial function.

Several limitations should also be acknowledged. First, follow-up duration was limited and dementia has a long prodromal period, which may reduce our ability to capture long-term effects and later-onset dementia cases. Because VaD often emerges after years of cerebrovascular injury accumulation, short-term event numbers are limited and estimates may be imprecise; the restricted time window also limits our ability to fully exclude influences of preclinical metabolic abnormalities, detection bias, or reverse causation. Future work with longer follow-up and larger samples is needed to validate these findings and explore underlying mechanisms. Second, as an observational study, causal inference is limited and residual confounding cannot be excluded. Treatment-stratified findings may also be influenced by health behaviours and healthcare access; although multiple confounders were adjusted for, unmeasured factors may remain. Finally, participants were drawn from a single city, and external generalisability should be evaluated in other regions and populations.

## 5 Conclusion

Incident T2D was associated with increased subsequent risk of dementia, particularly AD, and the strength of this association varied by age at diagnosis, treatment status, and genetic and mitochondrial backgrounds. The findings indicate an association between timely metabolic management after T2D diagnosis and lower subsequent cognitive risk, warranting confirmation in studies with stronger causal inference. Incorporating multidimensional biomarkers such as PRS and mtDNA-CN into individualised risk stratification may facilitate identification of higher-risk midlife subgroups and inform future research on targeted risk reduction.

## 6 Contributors

These authors contributed equally: Wanqing Dong,Qibin Yuan,Benrui Wu.Wanqing Dong: Conceptualization; Data curation; Formal analysis; Methodology; Visualization; Roles/Writing - original draft; and Writing - review & editing. Qibin Yuan: Conceptualization; Data curation; Formal analysis; Roles/Writing - original draft; and Writing - review & editing. Benrui Wu: Conceptualization; Data curation; Formal analysis; Roles/Writing - original draft; and Writing - review & editing.Shiteng Gao: Conceptualization; Data curation; Roles/Writing - original draft; and Writing - review & editing.

## Funding

Guangzhou National Laboratory (GZNL2024A01001), Henan Province-Ministerial Joint Project (SBGJ202301010). The funder had no role in study implementation and interpretation of findings.

## Ethics approval

This study was conducted in compliance with all relevant regulations regarding the use of human study participants and was approved by the Ethics Committee of the First People’s Hospital of Kunshan (approval No. IEC-C-007-A07-V3.0). All participants provided written informed consent.

## Declaration of Generative AI and AI-assisted technologies in the writing process

Not applicable.

## Data sharing statement

The KARE cohort data remains confidential under privacy regulations, but research collaboration requests may be submitted to the corresponding authors.

## Declaration of competing interests

None declared.
